# Circulation of West Nile Virus and Usutu Virus in Europe: Overview and Challenges

**DOI:** 10.3390/v16040599

**Published:** 2024-04-12

**Authors:** Yannick Simonin

**Affiliations:** Pathogenesis and Control of Chronic and Emerging Infections, University of Montpellier, INSERM, EFS, 34000 Montpellier, France; yannick.simonin@umontpellier.fr

**Keywords:** arboviruses, West Nile Virus, Usutu Virus

## Abstract

West Nile Virus (WNV) and Usutu Virus (USUV) are both neurotropic mosquito-borne viruses belonging to the *Flaviviridae* family. These closely related viruses mainly follow an enzootic cycle involving mosquitoes as vectors and birds as amplifying hosts, but humans and other mammals can also be infected through mosquito bites. WNV was first identified in Uganda in 1937 and has since spread globally, notably in Europe, causing periodic outbreaks associated with severe cases of neuroinvasive diseases such as meningitis and encephalitis. USUV was initially isolated in 1959 in Swaziland and has also spread to Europe, primarily affecting birds and having a limited impact on human health. There has been a recent expansion of these viruses’ geographic range in Europe, facilitated by factors such as climate change, leading to increased human exposure. While sharing similar biological traits, ecology, and epidemiology, there are significant distinctions in their pathogenicity and their impact on both human and animal health. While WNV has been more extensively studied and is a significant public health concern in many regions, USUV has recently been gaining attention due to its emergence in Europe and the diversity of its circulating lineages. Understanding the pathophysiology, ecology, and transmission dynamics of these viruses is important to the implementation of effective surveillance and control measures. This perspective provides a brief overview of the current situation of these two viruses in Europe and outlines the significant challenges that need to be addressed in the coming years.

## 1. WNV and USUV: Two Closely Related Viruses

Environmental changes have a major impact on the emergence or re-emergence of infectious diseases [1]; thus, climate change and modifications of ecosystems resulting from biodiversity loss and changes in land use can represent environmental threats to human and animal health. Emerging infectious diseases, especially vector-borne diseases, are closely linked to changes in ecological processes influenced by such anthropogenic factors. Climate change, urbanization, and land use have an impact on vector dynamics, particularly mosquitoes, as well as on host reservoir populations and the transmission of pathogens by vectors. In recent decades, the number of emerging arboviruses (viruses transmitted through the bite of an infected arthropod) described worldwide has increased considerably. Certain arboviruses in particular have expanded their geographic range, resulting in an increased number of human outbreaks, thus representing an emerging threat for human health [2].

**Figure 1:**
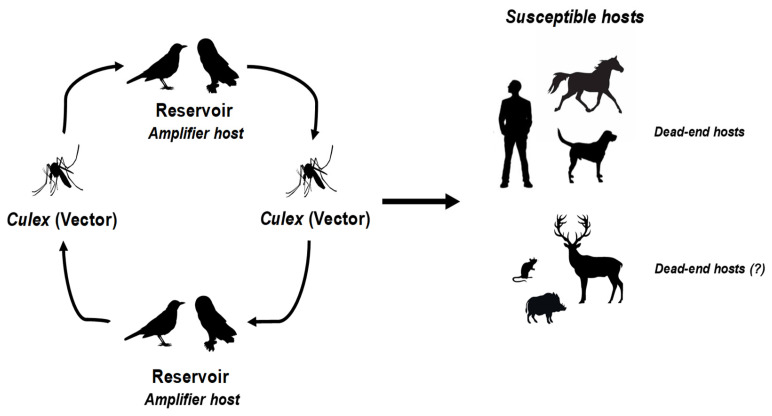
WNV and USUV transmission cycle involves birds (amplifying hosts) and mosquitoes (vectors). Infection can spread to humans and a diverse range of vertebrates, which are generally considered incidental or “dead-end” hosts.

Among them, West Nile Virus (WNV) and Usutu Virus (USUV) are two closely related neurotropic mosquito-borne viruses [3]. Both are enveloped, single-stranded RNA viruses belonging to the Japanese encephalitis virus serocomplex within the *Orthoflavivirus* genus, *Flaviviridae* family. WNV was first isolated from a woman in the West Nile district of Uganda in 1937, while USUV was first identified in Swaziland in 1959 from field-caught *Culex neavei* mosquitoes [4,5,6]. These viruses are maintained in similar transmission cycles involving ornithophilic mosquitoes, with resident or migratory birds acting as amplifying hosts (Figure 1).

On the European continent, the main vector of these viruses is the Culex pipiens mosquito [7,8,9]. Experimental vector competence has nevertheless been demonstrated by Aedes albopictus, as well as natural carriage [10,11,12]. USUV was also identified in *Aedes japonicus* japonicus mosquitoes [13]. Birds are currently considered the major factor in the spread of WNV throughout Europe, particularly through wetland areas such as the Po Delta in Italy, the Aliakmonas Delta in Greece, and the Rhône Delta in France. These marshy areas combine the parameters for a successful transmission cycle, being rich in mosquitoes; often harboring equine and human populations; and, above all, attracting a multitude of migratory birds which stop there to rest during migration. For example, the American robin Turdus migratorius appears to have been a major player in the dissemination of WNV on the American continent [14]. Furthermore, passerine birds, especially sparrows, are considered an important potential reservoir for WNV and USUV. The pathogenicity of WNV is particularly pronounced in certain avian species such as the European goshawk (*Accipiter gentilis*), which can die from the infection, whereas sparrows show lower mortality despite having high viremia [15,16]. Though they are considered reservoirs, some avian species are particularly sensitive to USUV infection, with a high mortality rate following infection. This is notably the case for Eurasian blackbirds (*Turdus merula*), great grey owls (*Strix nebulosa*), and house sparrows (*Passer domesticus*) [17,18]. Viral and necrotic lesions, notably neurological, have been identified in numerous organs of carcasses of these species. A significant die-off of birds, predominantly Eurasian blackbirds, across the European continent, notably in 2018, also underscores the potent pathogenicity of USUV among diverse avian populations [19,20,21,22]. Several avian species in particular, including the common kestrel (*Falco tinnunculus*) and the less common whitethroat (*Sylvia curruca*), may have contributed to the introduction of USUV into Europe, with Eurasian blackbirds, magpies, and sparrows subsequently spreading the virus across the European continent [23,24].

Natural occurrences of WNV and USUV have also been observed in over 100 mammalian species, including wild boars, wild ruminants, bats, rodents, and shrews [23,25,26,27,28,29,30,31,32]. It would, therefore, appear that mammals living in the wild are naturally exposed to these viral infections. Further investigation into their potential as hosts could shed light on their role in the virus transmission cycle. WNV and USUV infection in horses, like in humans, is considered to be an epidemiological dead end [33].

In humans, the incubation period of WNV is approximately 3 to 6 days, with a maximum range of 2 to 15 days, following a bite by an infected mosquito [34]. Approximately 20 to 30% of cases are symptomatic. The most common symptoms include fatigue and headaches, associated with flu-like symptoms which are referred to as “West Nile Fever” (WNF) [35]. It is estimated that approximately 1% of cases are characterized by West Nile neuroinvasive disease (WNND), such as encephalitis, meningitis, meningoencephalitis, or acute flaccid paralysis [35]. In instances of neuroinvasive complications, the mortality rate stands at around 10%, primarily affecting vulnerable individuals such as elderly or immunocompromised patients [36,37]. Severe sequelae can persist in 20 to 40% of individuals who survive [36]. For USUV, the small number of human cases that have been characterized thus far offer a less comprehensive understanding of the associated pathogenesis compared to WNV. Seroprevalence studies indicate that the majority of cases of USUV are asymptomatic or associated with only mild symptoms. The symptomatic phase of the infection has been described as possibly featuring moderate fever, along with a skin rash or febrile jaundice [38]. In a small number of cases, severe neurological complications may occur, such as meningitis, encephalitis, or meningoencephalitis [39]. It is worth noting that severe complications most often manifest in immunocompromised patients, but have occasionally been described in healthy individuals [40].

## 2. Expansion of the Distribution Range of These Viruses in Europe

The geographical distribution of WNV in Europe has expanded considerably over the past two decades. Europe’s first WNV epidemic occurred in 1996 in Romania, with 835 detected cases, including 17 deaths [41]. This was followed by cases of meningoencephalitis associated with WNV infection in Russia in 1999, where approximately 40 deaths were recorded [42]. The circulation of WNV also affects avian and equine populations, with significant epizootics, including the 220 cases of equine infection reported in France in 2000 and the significant bird mortality reported in 2018 and 2019 [43]. Between 2012 and 2021, a total of 3632 autochthonous cases of WNV infection in humans were reported in Europe [44].

USUV was first identified in Austria in 2001 [45] and retrospectively in Italy in 1996 [46], where it was linked to the deaths of a significant number of birds, including blackbirds [47]. From that point on, USUV began to be detected sporadically in animals (mainly birds) and mosquito vectors in several European countries (Germany, Belgium, Spain, France, Hungary, Italy, the Czech Republic, and Switzerland), suggesting its endemicity in those areas [39,40,48]. In both 2016 and 2018, new epizootics occurred in multiple European countries, among them, Austria, Belgium, France, Germany, Hungary, and the Netherlands [39]. Regarding cases in humans, to date, over 100 cases of acute human infection have been reported in Europe, including approximately 30 patients with neurological symptoms [40].

**Figure 2:**
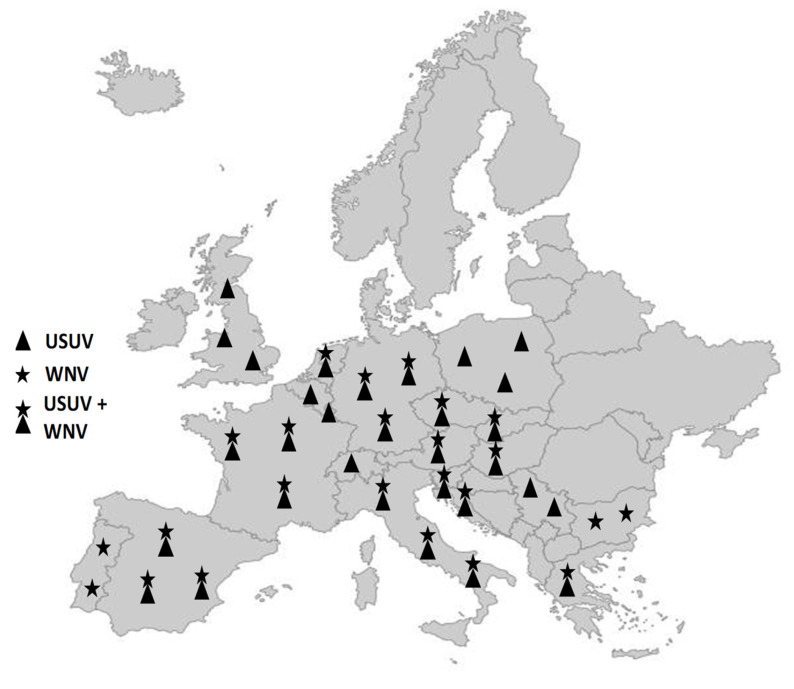
Distribution of WNV and USUV in Europe. Countries reporting only USUV: Belgium, Luxembourg, Poland, Serbia, Switzerland, United Kingdom. Countries reporting only WNV: Bulgaria, Portugal. Countries reporting both WNV and USUV: Austria, Croatia, the Czech Republic, France, Germany, Greece, Hungary, Italy, Netherlands, Slovakia, Slovenia, Spain.

While WNV and USUV were not considered endemic in Europe until the 2010s, recent years have seen epidemiological patterns of these viruses in several European regions that call for an urgent, in-depth analysis of their endemicity [49]. Over the past decades, WNV and USUV have been periodically detected in Europe, being associated mainly with sporadic cases in humans, horses, and birds, and, until recently, being limited to certain European regions [50]. For example, the occasional outbreaks of WNV over the past two decades have tended to take place in Central and Southern Europe [16,51,52,53,54]. The geographical distribution and intensity of WNV and USUV outbreaks in recent years, however, suggest a change in the epidemiological situation in Europe. Presently, epidemiological trends in various European regions indicate the endemic presence of these viruses, rather than merely periodic introductions from endemic areas. For instance, the recurrent detection of identical strains of WNV in the same region over several consecutive years has been confirmed in Northern Italy, Southern France, Hungary, and Austria [48,49,55,56]. WNV has also begun to affect higher latitudes within Europe, while the number of countries with USUV outbreaks is rapidly increasing [16,57,58,59,60,61,62] (Figure 2).

In 2018, the largest European WNV epidemic took place, with 2083 cases and 181 recorded deaths, representing more human cases than in the previous seven years [34,39,44,63,64]. This epidemic was concomitantly associated with a major USUV epidezootic, which affected several European countries and caused massive mortality in several bird species [39]. The year 2022 was another year of intense WNV circulation, as exemplified by Italy, where a season of dramatic transmission levels (over 500 WNV-positive cases, with over 50 deaths) was symptomatic of the disease’s high endemicity in certain European countries [65]. In 2023, the geographical expansion of these viruses continued, with 707 human cases of WNV being identified, mainly in Italy, Greece, and Romania. Cases of WNV infection were seen in several regions for the very first time, as were cases of USUV infection, albeit to a lesser extent. This was the case in the Nouvelle Aquitaine region of France [66,67].

**Figure 3:**
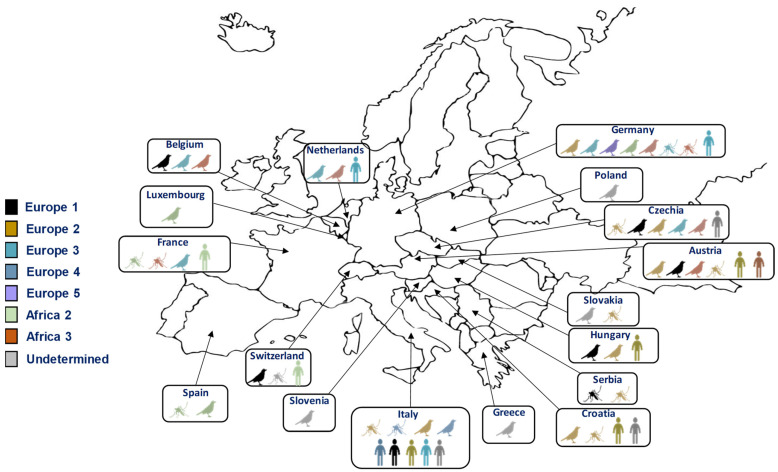
Distribution of different lineages of USUV in Europe in humans, mosquitoes, and birds.

Alongside the geographic spread of these viruses, the diversity of circulating lineages and strains must also be considered. WNV exhibits significant genetic diversity, encompassing at least nine distinct lineages [68]. Among these, two major lineages (lineages 1 and 2) have been responsible for the human outbreaks observed in Europe in recent years [69]. Lineages 3, 4, 5, 6, 7, 8, and 9 are represented only by a few isolates. Lineage 1 was responsible for the highest number of human and equine infections in Europe until 2004, and is also the lineage that has spread in North and Central America [70]. Lineage 2, which had been circulating in avian populations, first appeared in the human population in Hungary, and has more recently spread throughout Europe and the Mediterranean region [71]. It is probable that USUV, like WNV, spread from Africa to Europe through the migrations of its avian reservoir host. These viral circulations explain the multiple introductions of the virus into Europe from Africa and the different lineages that have emerged, which are also linked to the transmission cycle. Eight lineages have been delineated for USUV strains, named according to the origin of the first isolated strain (i.e., three African and five European lineages) [72]. All lineages, except the Africa 1 lineage, have been detected in Europe [72] (Figure 3). Although the majority of circulating strains currently in Europe are of European lineages, it is probable that African lineages continue to be introduced onto the continent, such as the Africa 2 and 3 lineages, which were discovered in *Culex* mosquitoes in France and Germany in 2018 [73,74,75].

## 3. Main Challenges to Overcome

### 3.1. Diagnostic Tools

Typically, the diagnosis of a WNV or USUV infection involves identifying specific antibodies or detecting the viral genome in whole blood; serum; or, occasionally, cerebrospinal fluid (CSF). However, acknowledging the narrow diagnostic window and low viral levels in the blood, the molecular testing of urine samples may broaden detection at a later stage. To date, viral detection in urine has been performed on WNV patients, on mice experimentally infected with USUV, and on USUV patients showing neurological symptoms. However, further studies are required to evaluate the routine utility of molecular virus detection [76,77]. Direct diagnosis hinges on detecting viral RNA in blood and CSF using a reverse-transcription polymerase chain reaction (RT-PCR). Because of the limited detection timeframe available for molecular tests, most human infections are diagnosed using serological methods. The most commonly used serological methods for detecting arbovirus-specific antibodies include enzyme-linked immunosorbent assays (ELISA) and neutralization tests. Distinguishing between the two infections poses a challenge, however, especially when serological methods are employed. While commercial ELISA assays are available and are relatively quick and inexpensive, they lack specificity in distinguishing between WNV and USUV due to a high degree of serological cross-reactivity. This cross-reactivity arises from similarities in their antigenic structures, with both their envelope and non-structural 1 antigens sharing a high amino acid homology. It is, therefore, necessary to perform virus-specific microneutralization tests, such as plaque reduction neutralization tests (PRNT), to confirm positive cases and to discriminate between WNV and USUV. While these microneutralization tests are more specific, they are time-consuming and can also produce cross-reactions [78].

Because of the constrained diagnostic capabilities and challenges in distinguishing between WNV and USUV infections, it is likely that numerous human USUV infections have gone undiagnosed or been incorrectly diagnosed as WNV infections [79]. An important hurdle in diagnosing USUV infection in humans has been the limited availability of validated commercial tests. Diagnosis in European countries has usually relied on internally developed laboratory methods [67]. The lack of homogeneity in the molecular or serological tests used in Europe to identify these viruses makes the data on their circulation less consistent. Therefore, research efforts should prioritize the development of specific serological tests to accurately distinguish between flavivirus infections. This is crucial to gain a deeper understanding of the epidemiology of these two viruses. It is also necessary to monitor and identify circulating strains and lineages. It is known, for example, that WNV lineages 1 and 2 are especially responsible for epidemics in humans. The presence of multiple circulating lineages of USUV emphasizes the necessity of monitoring the emergence of any new strains, some of which might exhibit heightened pathogenicity for humans [80,81]. It is also important to assess the potential implications of the co-infection or sequential infection of the two viruses and their different strains for existing diagnostic capabilities.

### 3.2. Blood and Organs Safety

Despite WNV causing only a short-lived viremia in humans, the potential viral exposure of donors in endemic regions is now acknowledged as a critical threat to transfusion safety. The virus can be efficiently transmitted even in donations from recently infected donors in whom virus concentrations are extremely low. Being the arbovirus with the most reported cases of transfusion transmission, particularly in the USA [82], WNV has significant implications for blood safety and security. Testing for WNV infection has been conducted on stem cell, tissue, and organ donations in several European countries where WNV is endemic. In the majority of cases, precautionary measures against WNV transmission were implemented during the transmission season and/or were prompted by the identification of the first human case. Only one case of WNV transmission from a single donation to two recipients has been documented in the EU, indicating that the existing blood safety measures effectively block WNV-infected blood donations from entering the EU blood supply [83]. However, the expanding distribution of WNV calls for a broader screening for the virus’ presence in blood products, and could lead to difficulties in managing blood donations in the most exposed areas.

Molecular and serologic surveillance in several European countries has identified USUV infections in blood donors [40]. While USUV is currently circulating more extensively than WNV in many European countries, no transfusion-associated USUV infections have been reported to date. However, the prevalence of USUV among blood donors remains unclear, as countries with USUV, but without WNV circulation, are not mandated to screen blood donations for orthoflavivirus RNA. Although no transfusion-associated USUV infections have been reported thus far, further investigations on this issue must be conducted. The co-circulation of WNV and USUV in multiple EU countries, combined with the undetermined transfusion risk and clinical significance of human USUV infections, also requires additional attention [84]. Shifts in the epidemiology of established infections and the rising incidence of WNV and USUV infections may call into question the applicability of current EU legislation concerning blood, tissues, and cells. In nations where blood donations are screened for WNV only, health authorities must recognize that positive WNV screening outcomes could stem from USUV infections and require further differentiation.

### 3.3. Vaccines and Treatments

Despite these viruses’ potential impact on human and animal health, current management approaches remain limited. While numerous efforts are underway to develop specific or broad-spectrum treatments for arboviruses, vector control remains the predominant method of prevention. However, vector management has proven less effective for WNV and USUV compared to arboviruses transmitted by *Aedes albopictus*, such as ZIKV or DENV. This is primarily due to the presence of animal reservoirs for these viruses, making vector control less suitable. Currently, there are no targeted treatments available for WNV or USUV in humans. Supportive symptomatic treatments, such as the use of paracetamol for pain and/or fever, hydration (oral or, in some cases, intravenous), and antiemetics (medication to alleviate vomiting and nausea), may be employed. The monitoring of vital signs such as intracranial pressure and respiratory rate, along with recommendations for rest, are also advised for particularly vulnerable patients.

While inactivated and recombinant vaccines for WNV are available for horses, the vaccination rate remains low [85,86]. Human vaccines against WNV have been developed, but none have yet undergone phase III clinical trial evaluation [87]. There are no authorized vaccines against USUV infection. One study details the protective impact of a recombinant DNA vaccine against lethal USUV challenge in an alpha/beta interferon receptor-deficient mouse model, and another documents the protective effects of an attenuated WNV-dengue virus 2 chimeric vaccine [88,89]. The development and approval of human vaccines face several limitations, including economic considerations, particularly for USUV, given that it has been implicated in only a limited number of human cases. Those limitations also include safety concerns, particularly the potential risk of antibody-dependent enhancement (ADE) with other orthoflaviviruses, such as Zika Virus or Dengue Virus [90]. The question of potential ADE effects or cross-protection also arises between WNV and USUV, notably due to their phylogenetic proximity and the fact that they co-circulate in the same geographic regions. Several studies have investigated the effect of cross-immunity between theses two viruses. Notably, the protective role played by USUV immunization before challenge with a lethal WNV strain has been demonstrated [91,92]. Moreover, a chimeric virus carrying the E protein of USUV in the WNV genome has shown an attenuated profile in mice compared to wild-type WNV [93]. Another study demonstrated that previous exposure to an attenuated WNV vaccine protects mice from a lethal USUV challenge [89], while the vaccination of adult mice with WNV-recombinant subviral particles induces low detectable levels of circulating IgG cross-reactive with USUV [94]. Interestingly, a study of pre-existing Usutu Virus immunity patients identified the presence of five atypical cases of WNV infection, characterized by the presence of WNV RNA and WNV IgG at the time of diagnosis, but found no IgM response during follow-up [95]. These data remain partial, however, and were typically conducted on murine models lacking an interferon response. In addition, the effect of different viral lineages must also be investigated, particularly as they exhibit differential virulence. Furthermore, studies must factor in the notion of cross-immunity in avian models, which are the main reservoirs of these viruses. Further investigation involving a larger number of cases is necessary to more precisely delineate the clinical and virological characteristics of WNV and USUV infection in individuals with pre-existing flavivirus immunity. One of the focuses of such an investigation should be to determine whether USUV infection offers cross-protection against WNV disease and vice versa or potentially increases the risk of more severe illness through antibody-dependent enhancement.

### 3.4. Development of Local Networks for One Health Approaches

The coexistence of WNV and USUV in both space and time presents considerable hurdles for surveillance and control efforts. It is difficult to precisely assess the impact of changing climate conditions, including alternating periods of drought, heavy rainfall, and conséquent flooding, on the epidemiology of WNV and USUV infection [96]. This is especially true when considering the potential effect on bird migration, the abundance and dynamics of vectors, and virus replication within these vectors, all of which may vary with temperature fluctuations. This underscores the necessity for enhanced monitoring and surveillance programs. While WNV and USUV share similarities in biology, ecology, and epidemiology, significant differences exist in their pathogenicity and their effects on human and animal health. These differences require comprehensive study, which necessitates the establishment of local surveillance networks. Such surveillance initiatives would require prompt identification and swift reactions from competent authorities. Augmenting surveillance programmes and improving diagnostic capabilities are key to the prompt and effective detection and management of epidemics or situations with epidemic potential. This requires the implementation of programs grounded in “One Health” principles if the spread of these viruses is to be properly appreciated and addressed. That framework acknowledges the interconnectedness of human health, animal health, and environmental health. This involves implementing strategies that rely on entomological, veterinary, and human surveillance activities. The virus was detected in mosquitoes and birds before any cases were reported in humans or horses. An integrated “One Health” surveillance system involving mosquitoes and birds across multiple European countries has already demonstrated its utility in the early detection of WNV/USUV circulation. This is notably exemplified in Italy, which serves as a model for organizing an integrated network for monitoring WNV and USUV viruses.

An analysis of the epidemiological landscape in Europe is crucial for devising effective surveillance strategies. That analysis is complex, however, due to various factors inherent in the epidemiology of WNV and USUV. An accurate assessment of their prevalence would require comprehensive surveillance across their diverse hosts, necessitating tailored sampling methods for humans, equids, wild birds, and mosquitoes. The fact that their outbreaks are not evenly distributed across Europe, with certain regions experiencing more frequent occurrences, would also have to be taken into account. In addition to this, virus dispersal via bird migration complicates any distinction between continuous presence and intermittent reintroductions, necessitating long-term studies to distinguish the two scenarios. It is also crucial that the viral strains circulating in Europe be studied more deeply to determine whether or not the same virus lineage persists over time, rather than a succession of different genotypes. Studying the potential viral persistence across seasons via mosquito overwintering is also essential. Ongoing research could offer new insights into the strains of WNV and USUV circulating in Europe, identifying potential molecular indicators of host specificity and virulence, as well as the consequences of their co-circulation in shared host and vector populations. Obtaining virus sequences or viral strains is challenging, however, due to the typically low prevalence of WNV and USUV in their respective host populations and the difficulty in accessing molecular samples of these viruses.

To date, the surveillance of WNV and USUV in birds has primarily focused on species found dead or easily captured and monitored; thus, most European countries currently rely on passive surveillance (i.e., on found dead birds). Given that not all species die from infection, however, more active programs (i.e., entomological or using animal sentinels) must be developed to study these viruses’ circulation in wild bird populations. However, the specific role of different bird species in maintaining the epidemiological cycle and transmitting these viruses to humans remains largely unknown. Similarly, the role of different mosquito species in the transmission of these viruses has not been well defined, with a notable lack of investigation into their individual vector competence for each of the two viruses, as well as in the context of co-infection. Indeed, the question of possible (albeit rare) competition between these two viruses must also be raised, especially in the case of co-infection within avian vectors and hosts [97]. Co-infections of arboviruses in mosquitoes may lead to an increase or a reduction in the transmission levels of one or both viruses. While several existing studies propose that USUV is disadvantaged by WNV in mammalian, avian, and mosquito cells during co-infection, further research on this point is required, preferably invoving a larger number of studied strains [98,99].

To conclude, WNV and USUV are now endemic in several countries in Southern Europe, with a gradual increase in cases detected in geographic areas further north. These viruses have many similarities, but also significant differences, all of which need to be better understood. Be it sequential or simultaneous, their co-circulation in an increasing number of geographic areas raises important questions and challenges. Indeed, the ecological niches of WNV and USUV overlap in many regions, leading to competition and interaction between them within mosquito vectors and avian hosts. To predict both viruses’ transmission patterns, assess the risk of spillover events to humans and animals, and develop effective control strategies, the genetic diversity, evolutionary dynamics, and ecological interactions of WNV and USUV populations must be better understood. Achieving this requires the application of integrated approaches, using adapted and sized surveillance networks for the simultaneous monitoring of both viruses, as well as further fundamental research into the pathogenicity and transmissibility of the wide variety of circulating strains.
